# Immune responses in COVID-19 patients during breakthrough infection with SARS-CoV-2 variants Delta, Omicron-BA.1 and Omicron-BA.5

**DOI:** 10.3389/fimmu.2023.1150667

**Published:** 2023-07-13

**Authors:** Maren Bormann, Leonie Brochhagen, Mira Alt, Mona Otte, Laura Thümmler, Lukas van de Sand, Ivana Kraiselburd, Alexander Thomas, Jule Gosch, Peer Braß, Sandra Ciesek, Marek Widera, Sebastian Dolff, Ulf Dittmer, Oliver Witzke, Folker Meyer, Monika Lindemann, Andreas Schönfeld, Hana Rohn, Adalbert Krawczyk

**Affiliations:** ^1^Department of Infectious Diseases, West German Centre of Infectious Diseases, University Hospital Essen, University of Duisburg-Essen, Essen, Germany; ^2^Institute for Transfusion Medicine, University Hospital Essen, University of Duisburg-Essen, Essen, Germany; ^3^Institute for Artificial Intelligence in Medicine, University Hospital Essen, Essen, Germany; ^4^Institute for Medical Virology, University Hospital Frankfurt, Goethe University Frankfurt, Frankfurt am Main, Germany; ^5^Institute of Pharmaceutical Biology, Goethe University Frankfurt, Frankfurt am Main, Germany; ^6^Fraunhofer Institute for Molecular Biology and Applied Ecology (IME), Branch Translational Medicine and Pharmacology, Frankfurt am Main, Germany; ^7^Institute for Virology, University Hospital Essen, University of Duisburg-Essen, Essen, Germany

**Keywords:** SARS-CoV-2, breakthrough infections, Omicron, Delta, COVID-19

## Abstract

**Background:**

Breakthrough infections with severe acute respiratory syndrome coronavirus 2 (SARS-CoV-2) variants are increasingly observed in vaccinated individuals. Immune responses towards SARS-CoV-2 variants, particularly Omicron-BA.5, are poorly understood. We investigated the humoral and cellular immune responses of hospitalized COVID-19 patients during Delta and Omicron infection waves.

**Methods:**

The corresponding SARS-CoV-2 variant of the respective patients were identified by whole genome sequencing. Humoral immune responses were analyzed by ELISA and a cell culture-based neutralization assay against SARS-CoV-2 D614G isolate (wildtype), Alpha, Delta (AY.43) and Omicron (BA.1 and BA.5). Cellular immunity was evaluated with an IFN-γ ELISpot assay.

**Results:**

On a cellular level, patients showed a minor IFN-γ response after stimulating PBMCs with mutated regions of SARS-CoV-2 variants. Neutralizing antibody titers against Omicron-BA.1 and especially BA.5 were strongly reduced. Double-vaccinated patients with Delta breakthrough infection showed a significantly increased neutralizing antibody response against Delta compared to double-vaccinated uninfected controls (median complete neutralization titer (NT_100_) 640 versus 80, p<0.05). Omicron-BA.1 infection increased neutralization titers against BA.1 in double-vaccinated patients (median NT_100_ of 160 in patients versus 20 in controls, p=0.07) and patients that received booster vaccination (median NT_100_ of 50 in patients versus 20 in controls, p=0.68). For boosted patients with BA.5 breakthrough infection, we found no enhancing effect on humoral immunity against SARS-CoV-2 variants.

**Conclusion:**

Neutralizing antibody titers against Omicron-BA.1 and especially BA.5 were strongly reduced in SARS-CoV-2 breakthrough infections. Delta and Omicron-BA.1 but not Omicron-BA.5 infections boosted the humoral immunity in double-vaccinated patients and patients with booster vaccination. Despite BA.5 breakthrough infection, those patients may still be vulnerable for reinfections with BA.5 or other newly emerging variants of concern.

## 1 Introduction

Since the beginning of the coronavirus disease 2019 (COVID-19) pandemic, more than 700 million people worldwide have been infected with severe acute respiratory syndrome coronavirus type 2 (SARS-CoV-2) and about seven million people have died as a result of COVID-19 (1). In an attempt to reduce the number of SARS-CoV-2 infections and severe COVID-19 cases, SARS-CoV-2 vaccines have been effectively deployed. The mRNA vaccines Comirnaty (BioNTech/Pfizer) and Spikevax (Moderna) have been administered most frequently in Germany, followed by Vaxzevria (AstraZeneca), Janssen (Johnson & Johnson) and Nuvaxovid (Novavax) (2). In particular, SARS-CoV-2 mRNA vaccines effectively protect against SARS-CoV-2 infection and severe COVID-19 (3, 4).

Throughout the COVID-19 pandemic, highly transmissible variants of concern (VOCs) have emerged, harboring multiple immune-escape mutations towards the available vaccines (5). By the end of 2021, the Omicron (B.1.1.529) variant displaced the Delta (B.1.617.2) variant as the leading VOC in Germany (6). Monoclonal antibodies as well as sera from vaccinated individuals are less effective in neutralizing Delta and Omicron compared to the D614G ancestral strain, with Omicron exhibiting the strongest immune evasiveness (7, 8). Despite the reduced neutralization capacity of vaccine-induced antibodies against these SARS-CoV-2 variants and the resulting increase of breakthrough infections among vaccinated individuals, most of the individuals with SARS-CoV-2 breakthrough infections were still protected against a lethal disease course (9–11). However, the humoral and cellular immune responses towards Omicron sub-variants BA.1 and in particular BA.5 are poorly understood.

In the present study, we assessed the humoral and cellular immune response in a group of patients hospitalized with SARS-CoV-2 breakthrough infection during Delta and Omicron infection waves. Our study sheds light on the extent of immune recall during breakthrough infection with Delta and Omicron-BA.1 and BA.5 in hospitalized patients and whether these infections provide a variant-specific immune boost or even cross-protective immunity.

## 2 Materials and methods

### 2.1 Study population

The study population consisted of 52 patients with a PCR-confirmed SARS-CoV-2 breakthrough infection hospitalized at the University Hospital Essen and a control group of 28 people without verified SARS-CoV-2 infection (**Table 1**). In total, 25 patients were infected with Delta, 15 with Omicron-BA.1 and 12 with Omicron-BA.5. The majority of Delta-infected patients were double vaccinated at the time of sample collection (88%). Patients with Omicron-BA.1 infection were predominantly double (53.3%) and triple (40%) vaccinated. All patients with Omicron-BA.5 infection was boosted, either with one booster dose (83.3%) or two booster doses (16.7%). Of the control group, 16 individuals were double vaccinated (57.1%), 10 were triple vaccinated (35.7%) and two were quadruple vaccinated (7.1%). Based on the definition of disease severity of COVID-19 by the World Health Organization (WHO), 42.2% of the patients had a non-severe course of COVID-19, 51.9% a severe course and 5.8% a non-severe course (12). Patient samples were collected from August 2021 to July 2022. Nasopharyngeal swabs and blood samples were collected to characterize the corresponding SARS-CoV-2 strain and the humoral and cellular immunity. Breakthrough infections were classified as Delta or Omicron based on sequencing information as well as information about infection waves from healthcare workers and patients at the University Hospital Essen (13).

**Table 1:** Overview of study cohort. Data indicate median (interquartile range) or absolute numbers (percentage).

Characteristics	Patients with Delta breakthrough infection (N=25)	Patients with Omicron-BA.1 breakthrough infection (N=15)	Patients with Omicron-BA.5 breakthrough infection (N=12)	Uninfected controls (N=28)	p
Sex:					
Men (%)	17 (68)	10 (66.7)	4 (33.3)	14 (50)	n.s.
Women (%)	8 (32)	5 (33.3)	8 (66.7)	14 (50)	
Age:					
Total	73 (51-82)	60 (55-77)	69 (62-79)	53 (49-63)	C-BA.5 and C-D: p<0.01
2 doses of vaccine	71 (49-83)	58 (44-63)	N/A	52 (48-64)	C-D: p=0.0457
Booster vaccination	71 (60-81)	74 (55-82)	69 (62-79)	54 (50-61)	C-BA.1 and C-BA.5: p<0.01
Vaccine:					
Comirnaty® (BioNTech/Pfizer) (%)	23 (92)	8 (53.3)	6 (50)	15 (53.6)	D-BA.1, D-BA.5 and C-D: p<0.01
Spikevax® (Moderna) (%)	1 (4)	2 (13.3)	0	12 (42.9)	C-BA.5 and C-D: p<0.01
Janssen® (Johnson & Johnson) (%)	1 (4)	0	0	0	n.s.
Combination (%)	0	5 (33.3)	5 (41.7)	1 (3.6)	D-BA-1, D-BA-5 and C-BA.5: p<0.01; C-BA.1: p<0.05
Unknown (%)	0	0	1 (8.3)	0	n.s.
Vaccine doses:					
1 (%)	1 (4)	1 (6.7)	0	0	n.s.
2 (%)	22 (88)	8 (53.3)	0	16 (57.1)	D-BA.1 and C-D: p<0.05; D-BA.5: p<0.0001; BA.1-BA.5: 0.01; C-BA.5: p<0.001
3 (%)	2 (8)	6 (40)	10 (83.3)	10 (35.7)	D-BA.1, BA.1-BA.5, C-D and C-BA.5: p<0.05; D-BA.5: p<0.0001
4 (%)	0	0	2 (16.7)	2 (7.1)	n.s.
Days since vaccination:					
Total	149.5 (97-184.3)	134.5 (66.25-192.5)	184.5 (133-222.5)	186 (45.75-199.5)	n.s.
Since 2nd vaccination	160 (113-188)	176 (90.5-229.3)	N/A	54 (29-186)	D-C: p=0.0414
Since booster	96.5 (91-102)	69 (46-140)	184.5 (133-222.5)	199 (192.3-208)	D-BA.5, BA.1-BA.5 and D-C: p<0.05; C-BA.1: p<0.01
Unknown	7	1	4	0	

Data indicate median (interquartile range) or absolute numbers (percentage). Differences between groups for the categorical variables were analyzed by Fisher’s exact test and for the continuous variables by two-tailed Mann-Whitney U test. N/A, not applicable; D, Delta; C, uninfected control; n.s., not significant.

The study was approved by the local ethics committee and was performed in accordance with the ethical standards noted in the 1964 Declaration of Helsinki and its later amendments or comparable ethics standards (approval no. 20-9665-BO). Informed consent was obtained from all participants in the study.

### 2.2 Cells and viruses

A549-AT cells were cultivated in minimum essential media (MEM), supplemented with 10% (v/v) fetal calf serum (FCS), penicillin (100 IU/mL) and streptomycin (100 µg/mL) at 37 °C in an atmosphere of 5% CO_2_ (all Life Technologies Gibco, Darmstadt, Germany) (14). These cells overexpress both the carboxypeptidase angiotensin I converting enzyme 2 (ACE2) receptor and the cellular transmembrane protease serine 2 (TMPRSS2), allowing for high SARS-CoV-2 susceptibility and formation of cytopathic effects (CPEs).

Nasopharyngeal swabs from COVID-19 patients were used to isolate variants of SARS-CoV-2 (15, 16). In brief, the swab medium was incubated on A549-AT cells for several days until a profound CPE became apparent. Subsequently, supernatant was harvested, cleared from cell debris by centrifugation and stored at -80°. Viral titers were determined using A549-AT cells by a standard end-point dilution assay and calculated as 50% tissue culture infective dose (TCID_50_)/mL as previously described (17).

### 2.3 Sequencing and phylogenetic characterization

SARS-CoV-2 RNA of cell culture supernatants and nasopharyngeal swabs was purified using the QIAamp Viral RNA Mini Kit (QIAGEN, Hilden, Germany). SARS-CoV-2 whole genome libraries were obtained with the EasySeq™ SARS-CoV-2 Whole Genome NGS Sequencing kit (Nimagen, Nijmegen, Netherlands) after cDNA generation from 5.5 µl of viral RNA with the LunaScript RT SuperMix Kit (NEB). Pooled and normalized libraries were sequenced on an Illumina MiSeq instrument employing the V2 chemistry (300 cycles).

Data analysis was conducted by the opensource pipeline UnCoVar (18). Briefly, UnCoVar performs a series of QC steps, initially attempts *de-novo* assembly with reference guided scaffolding to achieve full genome reconstruction. Alternatively, the genome of recalcitrant samples is generated via incorporation of observed mutations to the Wuhan reference genome using variants called with Freebayes (19), Delly (20) and Varlociraptor (21). The workflow subsequently uses Pangolin (22) for genome lineage calling and Kallisto (23) for read based matching to 24 (25).

After obtaining whole genome sequences, sub-sequences were extracted according to the observed genomic features of the Wuhan reference genomes. For the selected features, e.g., the spike (S) protein coding region, as well as for the whole genome, sequences were aligned [mafft] and phylogenetic trees were calculated [iqtree] to obtain the evolutionary correlations between the samples.

### 2.4 SARS-CoV-2 S and NCP ELISA

IgG antibodies against subunit 1 of the SARS-CoV-2 S protein (S1; Wuhan-Hu-1 isolate) and IgG and IgM antibodies against the nucleocapsid protein (NCP) were measured from patient sera with an enzyme-linked immunosorbent assay (ELISA) (Euroimmun Medizinische Labordiagnostika, Lübeck, Germany). A ratio between the absorbance of the sample and calibrator of <0.8 was regarded as negative, ≥0.8 to <1.1 borderline, and ≥1.1 positive.

### 2.5 Neutralization Assay on A549-AT cells

The neutralization capacity of serum samples against a SARS-CoV-2 clinical isolate from September 2020 with the D614G mutation (wildtype) as well as the variants Alpha (B.1.1.7), Delta (AY.43) and Omicron (BA.1 and BA.5) was analyzed. Additionally, the neutralizing capacity of sera from ten patients was investigated (patient 1, 6, 9, 10, 24, 27, 33, 45, 48, 52) towards their equivalent clinical isolate that caused the SARS-CoV-2 breakthrough infection in comparison to wildtype isolate.

Neutralization assays were conducted as described previously (26). Briefly, two-fold serial dilutions of patient sera (1:20 to 1:2560) were pre-incubated with 100 TCID_50_/50 µL SARS-CoV-2 for one hour at 37 °C. These mixtures were added to A549-AT cells and incubated for three days at 37 °C and 5% CO_2_. Cell cultures were stained with 0.5% crystal violet (w/v) (Roth, Karlsruhe, Germany), solved in 20% (v/v) methanol (Merck, Darmstadt, Germany) and evaluated for CPEs by transmitted light microscopy. The highest serum dilution at which none of the triplicate cultures displayed CPEs was defined as the complete neutralization titer (NT_100_).

### 2.6 ELISpot Assay for SARS-CoV-2 S and NCP

An IFN-γ enzyme-linked-immuno-spot (ELISpot) assay was conducted to evaluate the cell-mediated immune response to SARS-CoV-2, as described before (27, 28). Plates equipped with polyvinylidene difluoride (PVDF) membranes (MilliporeSigma™ MultiScreen™ HTS, Fisher Scientific, Schwerte, Germany) were activated with ethanol. Subsequently, plates were coated with 60 µL monoclonal antibodies against IFN-y (10 µg/mL of clone 1-D1K, Mabtech, Nacka, Sweden). After washing and blocking with 150 µL AIM-V® (Thermo Fisher Scientific, Grand Island, NY, USA) for 30 minutes at 37 °C, 250,000 peripheral blood mononuclear cells (PBMCs) in 150 µL of AIM-V® in the presence or absence of PepTivator® proteins (600 pmol/mL of each peptide, all Miltenyi Biotec, Bergisch Gladbach, Germany) were added. The NCP, S protein of Wuhan wildtype and selectively mutated regions of Alpha (B.1.1.7), Delta (AY.1) and Omicron (B.1.1.529) were incubated for 19 hours at 37 °C followed by washing. To detect captured IFN-y, 50 µL alkaline phosphatase conjugated monoclonal antibody against IFN-y (clone 7-B6-1, Mabtech) diluted 1:200 in PBS containing 0.5% bovine serum albumin (BSA) was incubated for one hour. Plates were washed again, and nitro blue tetrazolium/5-bromo-4-chloro-3-indolyl-phosphate was added. Spots were quantified using an ELISpot reader (AID Fluorospot, Autoimmun Diagnostika GmbH, Strassberg, Germany). Non-stimulated values were subtracted from stimulated values to obtain the SARS-CoV-2 specific spots. A spot increment of three was considered positive.

### 2.7 Statistical analyses

Statistical analyses and data visualization were conducted using GraphPad Prism 9.4.0 (San Diego, CA, USA) software. For continuous variables, the median and interquartile range were calculated. Significant differences were assessed using Kruskal-Wallis test with post-hoc Dunn’s test for multiple comparison, Mann-Whitney U test and Wilcoxon signed-rank test for analyses of more than two independent groups, two unpaired samples and two paired samples, respectively. Categorical variables were analyzed by Fisher’s exact test. Correlation coefficients were calculated using Spearman’s rank analysis. *P*-values <0.05 were considered significant.

## 3 Results

### 3.1 Sequencing and phylogenetic analysis of SARS-CoV-2 variants causing breakthrough infections

At the time of sample collection, all study participants had received at least one vaccine dose. Of the control group, 57.1% individuals were double-vaccinated, 35.7% were triple-vaccinated and 7.1% were quadruple-vaccinated. 88% of Delta-infected patients were double vaccinated. Of the patients with Omicron-BA.1 infection, 53.3% were double and 40% triple vaccinated. Omicron-BA.5 infected patients were all boosted with either one booster dose (83.3%) or two booster doses (16.7%).

**Figure 1:**
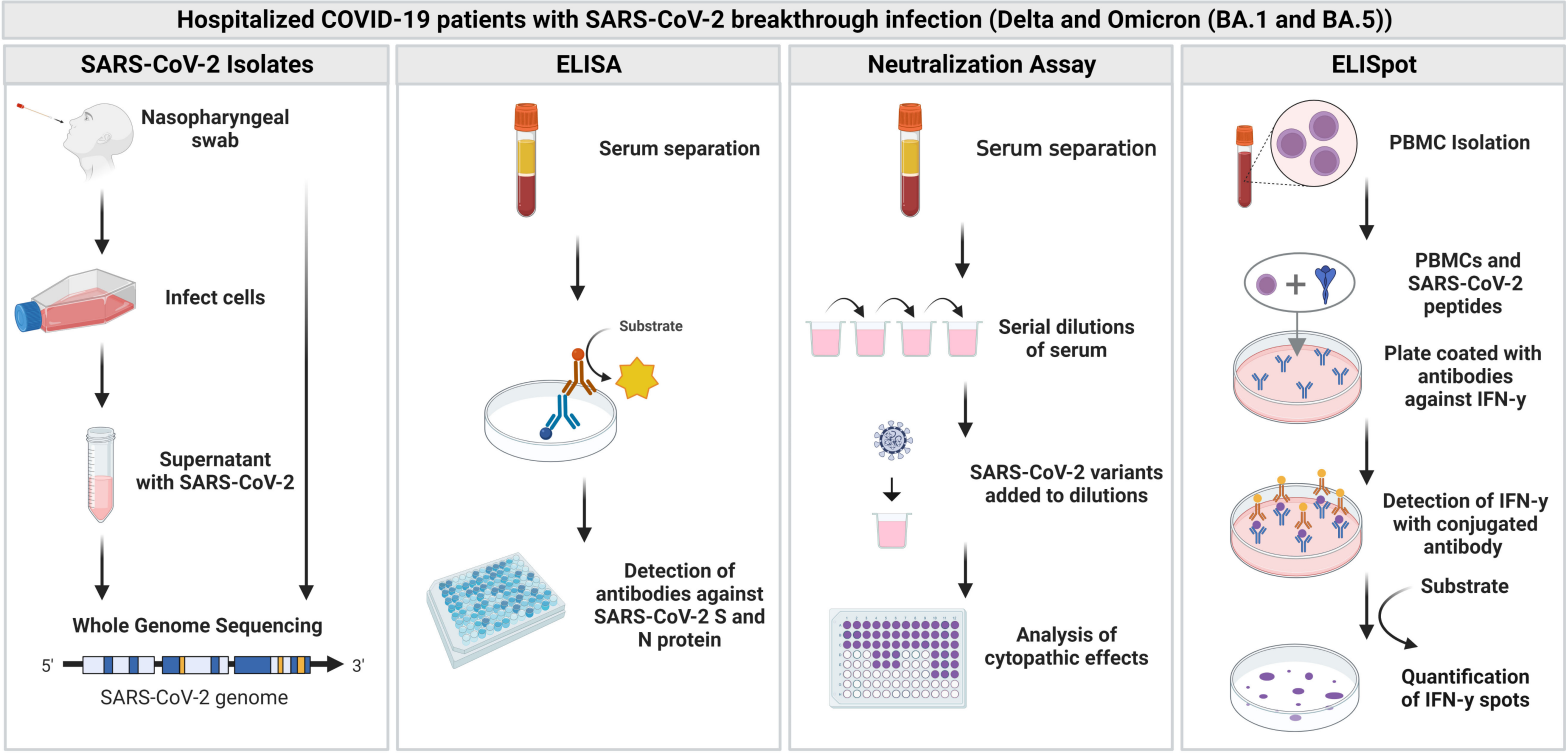
Overview of the study. Blood samples and nasopharyngeal swabs were collected from hospitalized patients with SARS-CoV-2 breakthrough infection (Delta and Omicron). Blood samples were further analyzed with an enzyme-linked immunosorbent assay (ELISA), neutralization assay as well as enzyme-linked-immuno-spot (ELISpot) assay. Figure was created with BioRender.com.

**Figure 2:**
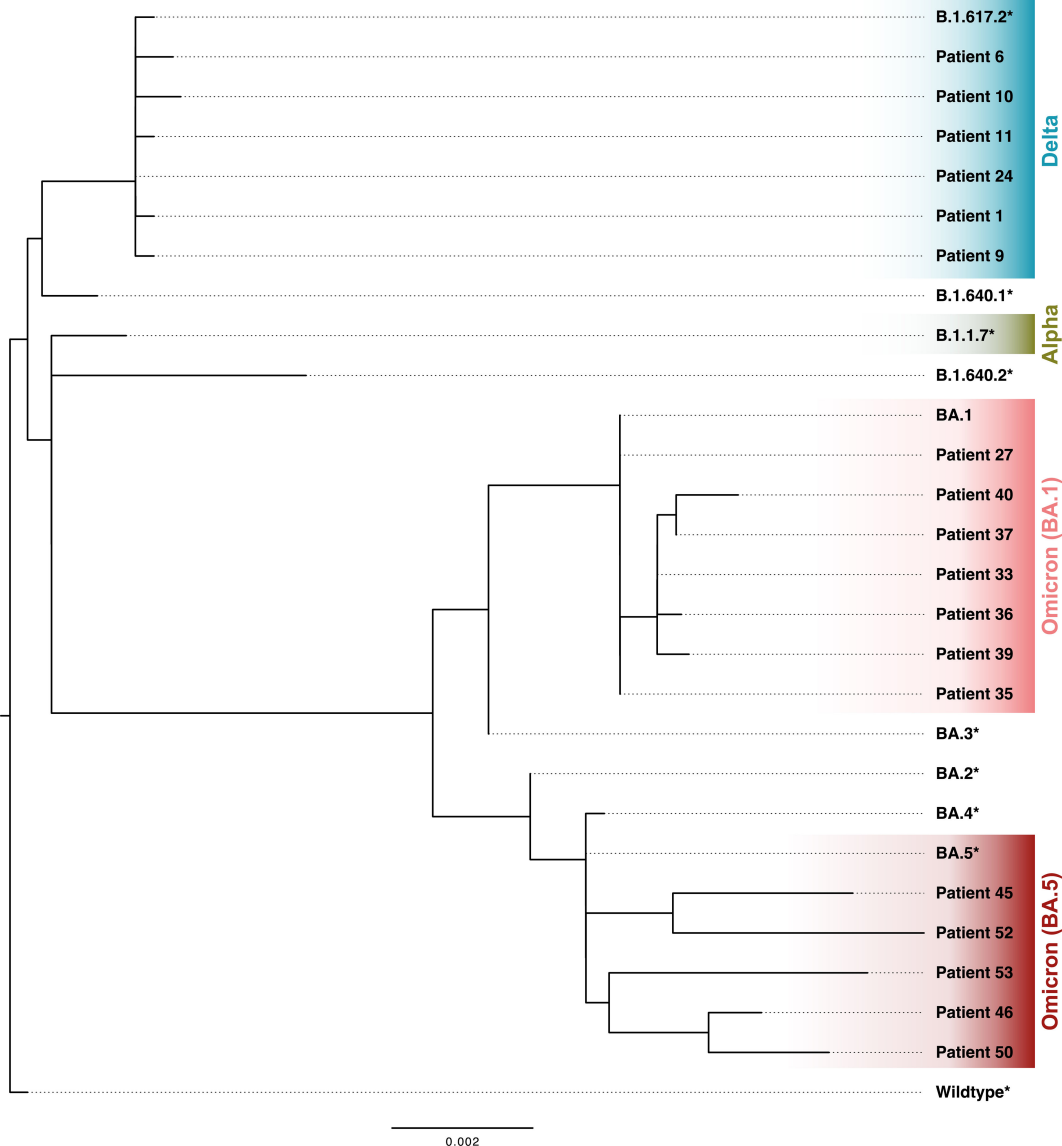
Phylogenetic tree of assembled SARS-CoV-2 spike (S) region sequences of clinical isolates of patients with SARS-CoV-2 breakthrough infection. * S region extracted from GISAID reference genomes (GISAID).

Clinical isolates of hospitalized patients with SARS-CoV-2 breakthrough infection were sequenced by whole genome sequencing (**Figure 1**). S region sequences were successfully assembled from 18 patients. These patients were infected with Delta (B.1.617.2) and Omicron sub-lineages BA.1 and BA.5 (**Figure 2**). The remaining patients were classified based on information about infection waves from healthcare workers and patients at the University Hospital Essen (13). The phylogenetic analysis highlights the continuous evolution of SARS-CoV-2, which poses a challenge for vaccine development.

### 3.2 SARS-CoV-2 binding serum antibody levels

**Figure 3:**
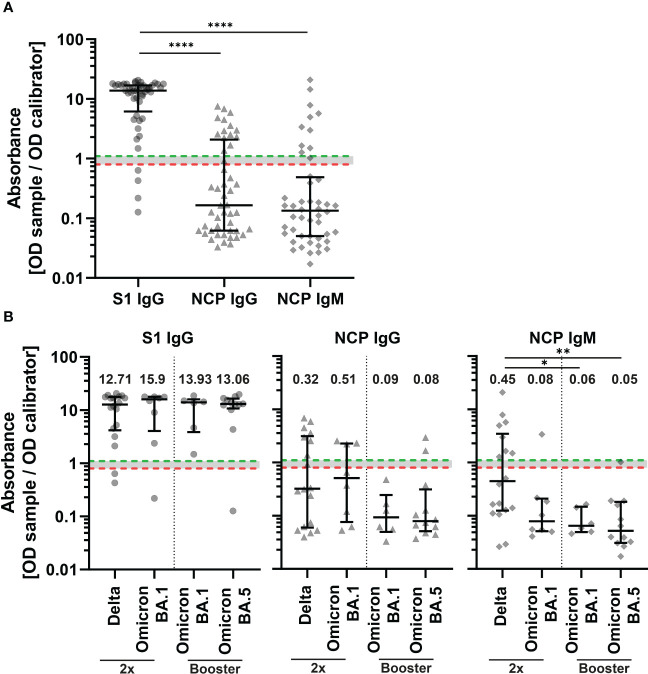
Binding serum antibody levels in COVID-19 patients with SARS-CoV-2 breakthrough infection. IgG antibodies against the subunit 1 of spike protein (S1) (Wuhan-Hu-1 isolate) and IgG and IgM antibodies against the nucleocapsid protein (NCP) of all patients (n=48) **(A)** and double vaccinated patients (2x) with Delta (n=18) and BA.1 (n=8) infection and patients with booster vaccination with BA.1 (n=6) and BA.5 (n=12) infection **(B)**. Binding serum antibodies were measured with an enzyme-linked immunosorbent assay (ELISA). An absorbance of <0.8 was regarded as negative (red dotted line), ≥0.8 to <1.1 borderline, and ≥1.1 positive (green dotted line). Differences between groups were analyzed by Kruskal-Wallis test with post-hoc Dunn’s multiple comparisons test (* p<0.05; ** p<0.01, **** *p<*0.0001). Horizontal lines indicate median values, while error bars represent the interquartile range.

Sera of patients with SARS-CoV-2 breakthrough infection were tested for SARS-CoV-2 subunit 1 (S1) specific IgG antibodies and IgG and IgM antibodies against the nucleocapsid protein (NCP) by an enzyme-linked immunosorbent assay (ELISA) (**Figure 1**). Overall, 91.7% of samples were positive for S1 specific antibodies (**Figure 3A**). Next, we measured IgM and IgG antibody levels against NCP of SARS-CoV-2 to distinguish between the early and late humoral responses during infection. Antibody levels against the NCP were significantly lower compared to S1 (p<0.0001) (**Figure 3A**). In total, 20.8% of patient sera were positive for IgM antibodies and 29.2% for IgG antibodies. When dividing patients by breakthrough infection and number of vaccines, there were no significant differences in S1 and NCP IgG levels between groups (**Figure 3B**). However, patients with Delta breakthrough infection who received two vaccine doses had significantly higher levels of NCP IgM compared to patients with booster vaccination and Omicron BA.1 infection (p<0.05) as well as patients with booster vaccination and Omicron BA.5 infection (p<0.01).

### 3.3 Neutralizing antibody titers in sera after Delta, BA.1 or BA.5 breakthrough infection

**Figure 4:**
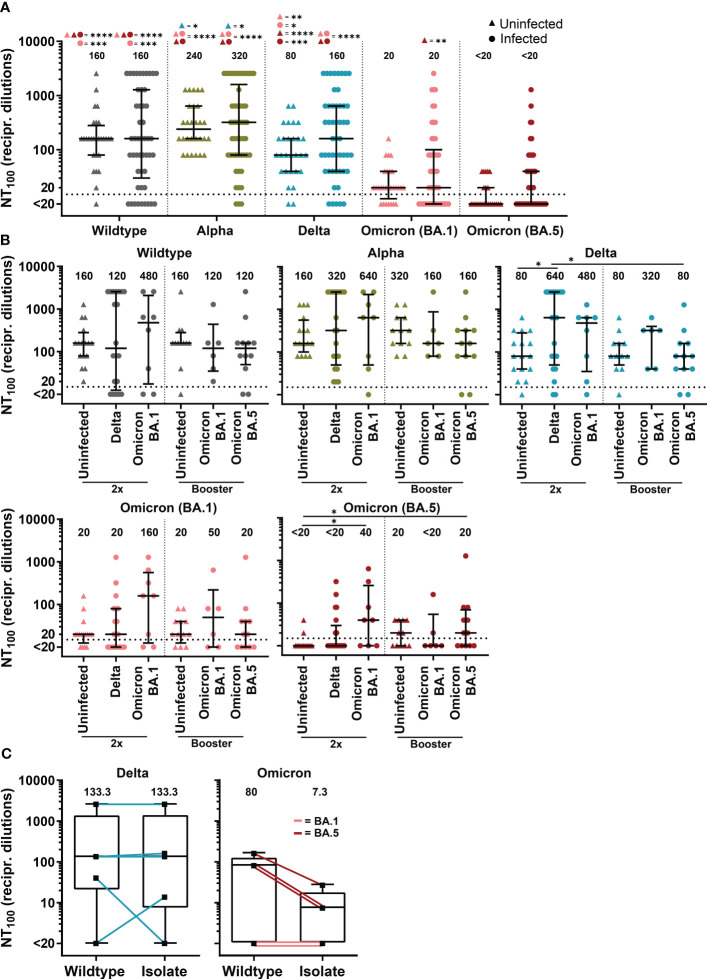
Neutralizing antibody titers against SARS-CoV-2 variants of COVID-19 patients with SARS-CoV-2 breakthrough infection and uninfected, vaccinated controls. **(A)** Complete neutralization titer (NT_100_) against clinical isolate with D614G mutation (wildtype), Alpha (B.1.1.7), Delta (AY.43) and Omicron (BA.1 and BA.5) of patients with SARS-CoV-2 breakthrough infection (n=50) compared to vaccinated uninfected control (n=28). **(B)** NT_100_ against clinical isolate with D614G mutation (wildtype), Alpha (B.1.1.7), Delta (AY.43) and Omicron (BA.1 and BA.5) of double vaccinated patients (2x) with Delta (n=20) and BA.1 (n=8) infection and patients with booster vaccination with BA.1 (n=6) and BA.5 (n=12) infection compared to uninfected control with two vaccine doses (n=16) and booster vaccination (n=12). **(A, B)** Differences between groups were analyzed by Kruskal-Wallis test with post-hoc Dunn’s multiple comparisons test (* *p<*0.05; ** *p<*0.01; *** *p<*0.001; **** *p<*0.0001). **(C)** NT_100_ of sera from patients with breakthrough infection with Delta and Omicron (BA.1 and BA.5) against their equivalent clinical isolate compared to wildtype. Differences between groups were analyzed by Wilcoxon signed-rank test. **(A–C)** Horizontal lines indicate median values, while error bars represent the interquartile range.

The humoral immunity of COVID-19 patients with SARS-CoV-2 breakthrough infections was further investigated using a cell culture-based neutralization assay. Serum samples from those patients were tested against a SARS-CoV-2 D614G wildtype clinical isolate and Alpha (B.1.1.7), Delta (AY.43) and the Omicron sub-lineages BA.1 and BA.5. Sera from COVID-19 patients as well as sera from non-infected but immunized individuals showed reduced complete neutralization titers (NT_100_) towards BA.1 and BA.5 compared to wildtype, Alpha and Delta (**Figure 4A**).

Double-vaccinated patients with Delta breakthrough infection displayed a significantly increased neutralizing antibody response against Delta compared to double-vaccinated uninfected controls (median NT_100_ 640 versus 80, p<0.05, **Figure 4B**). In double-vaccinated patients, infection with Omicron sub-lineage BA.1 boosted immunity against BA.1 just above statistical significance (median NT_100_ of 160 in patients versus 20 in controls, p=0.07) as well as against BA.5 (median NT_100_ of 40 in patients versus <20 in controls, p<0.05) (**Figure 4B**). A higher median NT_100_ against BA.1 was also observed for boosted Omicron-BA.1 infected patient compared to boosted controls (median NT_100_ of 50 versus 20, p=0.68). Interestingly, results suggest cross-reactive immunity for patients with Omicron-BA.1 infection against Delta, as double-vaccinated had a 6-fold (median NT_100_ of 480 versus 80, p=0.24) and boosted a 4-fold (median NT_100_ of 320 versus 80, p=0.44) higher NT_100_ than control. For individuals with BA.5 infection, we observed no immune boost against BA.5 or other variants.

Next, we investigated neutralization capacity of patient sera against the SARS-CoV-2 clinical isolate from these respective patients compared to wildtype. In total, ten different SARS-CoV-2 isolates from patients infected with sub-lineages of Delta and Omicron could be propagated in cell culture to investigate the respective neutralizing antibody titers. Patients infected with Delta showed similar neutralization efficacy against their isolate compared to wildtype (**Figure 4C**). In contrast, we found reduced neutralization capacity against isolates from Omicron-infected patients in comparison to wildtype (median NT_100_ of 7.3 versus 80, p=0.25).

In summary, we found that Delta infections exhibit a strong immune boosting effect against the Delta variant. Patients infected with BA.1 showed an increased neutralizing antibody response against both tested Omicron variants. Compared to Delta and BA.1, BA.5 was the least immunogenic variant, as BA.5 infections did not boost immunity against BA.5 or other variants.

### 3.4 Cellular immunity in patients with SARS-CoV-2 breakthrough infection

Cellular immunity against SARS-CoV-2 was measured using an IFN-γ enzyme-linked-immuno-spot (ELISpot) assay. We stimulated peripheral blood mononuclear cells (PBMCs) with the NCP, spike (S) protein of Wuhan wildtype and with selectively mutated regions of Alpha (B.1.1.7), Delta (AY.1) and Omicron (B.1.1.529). An IFN-γ-spots increment of three was considered positive.

**Figure 5:**
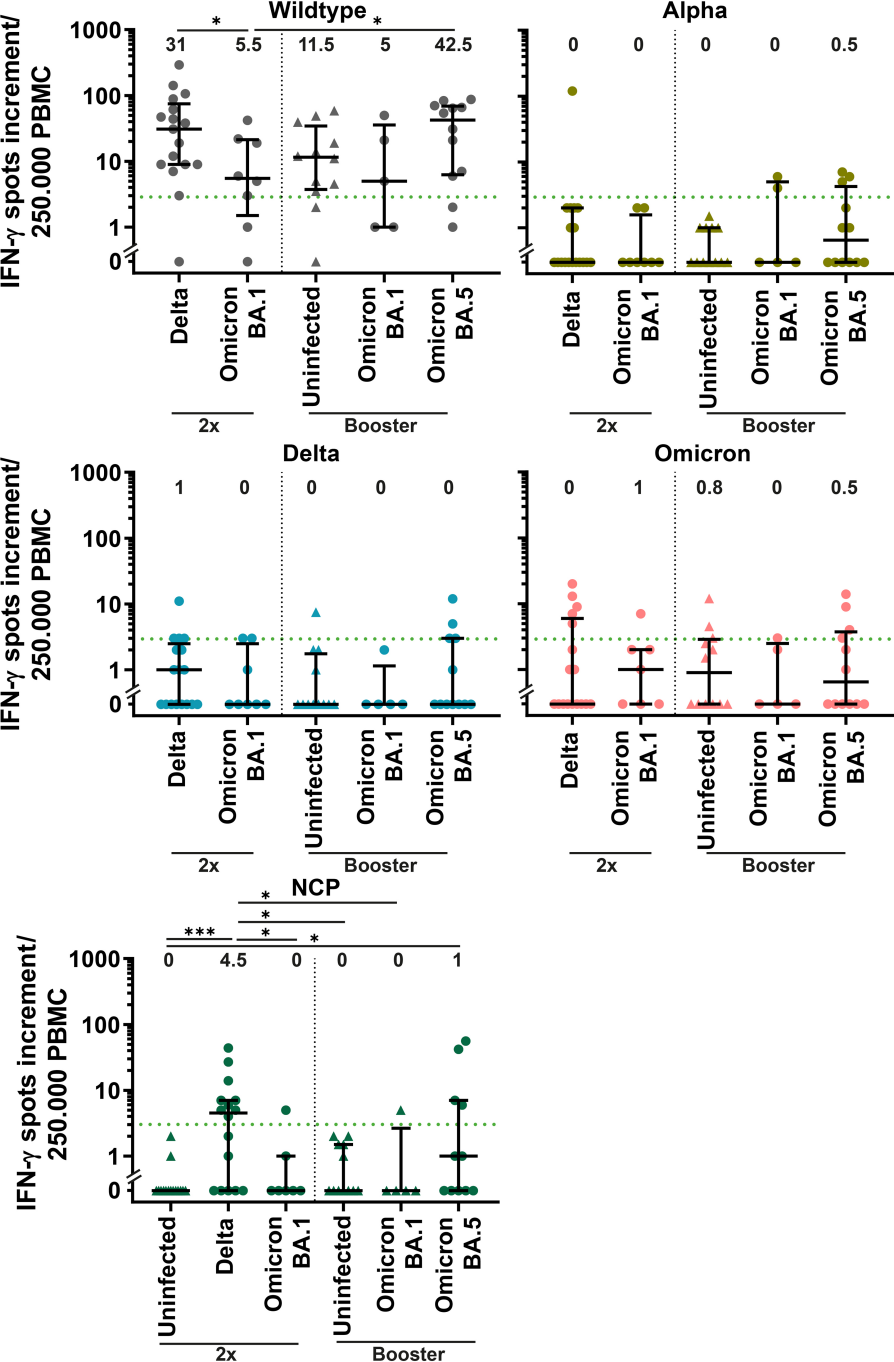
Cellular response against SARS-CoV-2 variants in COVID-19 patients with SARS-CoV-2 breakthrough infection. Cellular immunity was assessed by an IFN-γ enzyme-linked-immuno-spot (ELISpot) assay using peripheral blood mononuclear cells (PBMCs) and is displayed for double vaccinated patients (2x) with Delta (n=18) and BA.1 (n=8) infection and patients with booster vaccination with BA.1 (n=5) and BA.5 (n=12) infection compared to uninfected control with two vaccine doses (n=15) and booster vaccination (n=12). PBMCs were stimulated with S protein of Wuhan wildtype, nucleocapsid protein (NCP) and selectively mutated regions Alpha (B.1.1.7), Delta (AY.1) and Omicron (B.1.1.529). A spot increment of three was considered positive (green dotted line). Differences between groups were analyzed by Kruskal-Wallis test with post-hoc Dunn’s multiple comparisons test (* *p<*0.05; *** *p<*0.001). Horizontal lines indicate median values, while error bars represent the interquartile range.

Double-vaccinated patients with Delta infection showed the highest positivity in response to NCP stimulation, followed by boosted BA.5-infected patients (56.2% and 33.3%, respectively, **Figure 5**). As expected, infection-naïve participants did not show a positive NCP response. Among dually vaccinated patients, the IFN-γ spots increment was significantly higher for Delta-infected patients than for patients infected with BA.1 (31 versus 5.5, p<0.05) after stimulation with wildtype S protein. A significantly higher response to S wildtype was also observed for BA.5-infected patients compared to double-vaccinated BA.1-infected patients (42.5 versus 5.5, p<0.05). All groups showed a median IFN-γ spots increment below positivity to mutated regions of SARS-CoV-2 variants (**Figure 5**).

### 3.5 Correlation between SARS-CoV-2 ELISA IgG antibody levels and neutralizing antibody titers as well as cellular IFN-γ response

**Figure 6:**
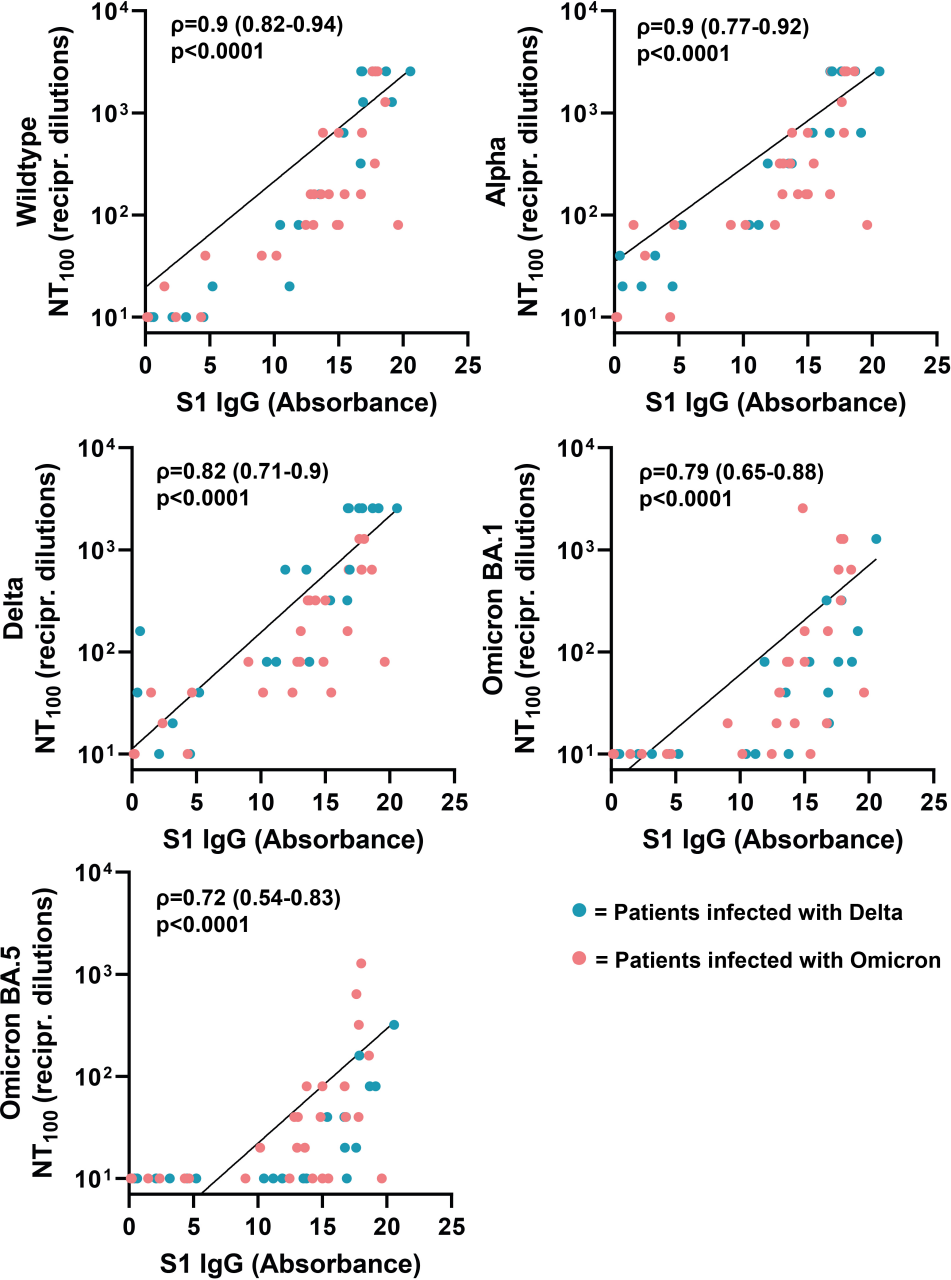
Correlation between serum antibody levels and neutralizing antibody titers against SARS-CoV-2 variants. Correlation coefficients (ρ) and *p*-values were calculated using Spearman’s rank analysis.

**Figure 7:**
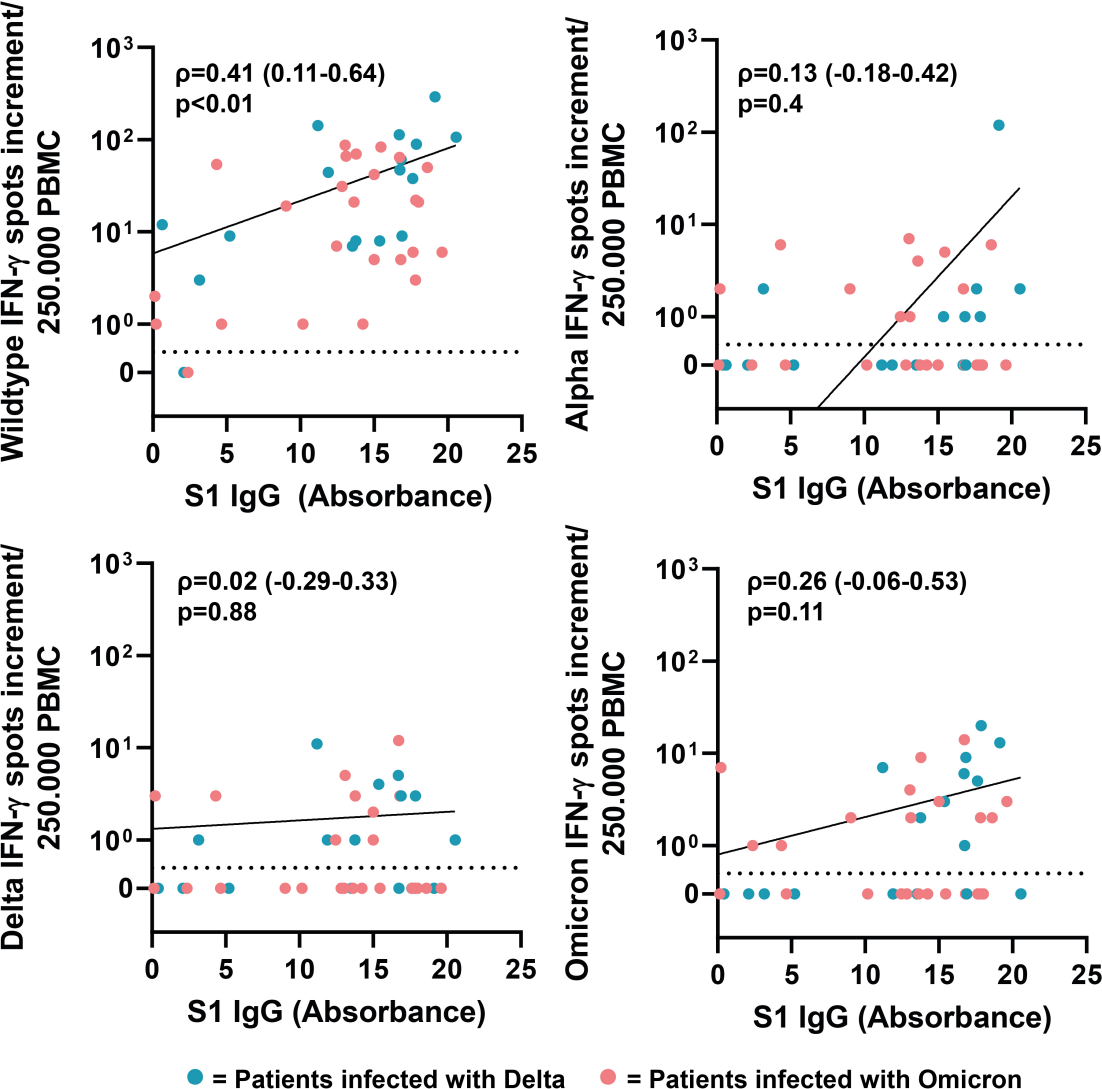
Correlation between serum antibody levels and cellular IFN-γ response after stimulation with SARS-CoV-2 variants. Correlation coefficients (ρ) and *p*-values were calculated using Spearman’s rank analysis.

Next, we analyzed if there is a correlation between neutralizing antibody titers of the respective sera against SARS-CoV-2 wildtype, Alpha, Delta and Omicron (BA.1 and BA.5) and SARS-CoV-2 ELISA IgG antibody levels against S1 (Wuhan-Hu-1 isolate). The neutralizing antibody titers correlated positively with ELISA IgG antibody levels (**Figure 6**). The highest correlation was observed for neutralizing antibody titers against wildtype and Alpha (Spearman’s ρ=0.9, respectively). Compared to wildtype and Alpha, we observed a lower correlation for Delta, Omicron-BA-1 and Omicron-BA.5, with Spearman’s rank coefficients of 0.82, 0.79 and 0.72, respectively. Next, we analyzed the correlation between IgG antibodies against S1 and cellular IFN-γ production in response to stimulation with SARS-CoV-2 variants. The results only revealed a correlation between SARS-CoV-2 ELISA IgG antibody levels and the cellular immune response against wildtype (ρ=0.41), but not SARS-CoV-2 variants (**Figure 7**).

## 4 Discussion

Throughout the COVID-19 pandemic, several SARS-CoV-2 variants with immune-escape mutations have emerged, leading to an increase of SARS-CoV-2 breakthrough infections (5). In the present study, we report on the humoral and cellular immunity in response to Delta and Omicron (BA.1 and BA.5) infection in a group of vaccinated patients with SARS-CoV-2 breakthrough infections. We compared the results to vaccinated uninfected controls, to assess the additive effect of the infection on immunity.

Of note, the neutralizing antibody titers against Omicron sub-variants BA.1 and especially BA.5 were strongly reduced when compared to Alpha, Delta or wildtype. These findings are consistent with recently published data using pseudovirus-neutralization assays, showing a substantial immune escape of BA.5 sub-variant against antibodies of vaccinated individuals or individuals infected with BA.1 or BA.2 (29–31).

In line with recent studies, we showed that Delta and Omicron-BA.1 infections lead to a strain-specific boost of neutralizing immunity (32, 33). Previous data indicated that Delta breakthrough infections increase Delta specific neutralization titers to levels comparable to wildtype neutralization (32). In our study, Delta infection markedly increased neutralizing antibody titers against Delta in double-vaccinated patients, even with a 5.3-fold higher neutralizing antibody titer against Delta compared to wildtype. Omicron-BA.1 breakthrough infection enhanced the neutralizing antibody titer against BA.1 and Delta (33). Notably, the neutralizing antibody titer of sera from uninfected controls was 8-fold reduced against BA.1 when compared to wildtype. In contrast, in double-vaccinated patients with BA.1 infection the ratio between BA.1 and wildtype neutralizing antibody titers reduced to 3 and in boosted patients to 2.4.

Additionally, our study provides insight into the immunity in BA.5 breakthrough infections. We found no evidence of a boosting effect on humoral immunity for this sub-variant, which could increase the likelihood of reinfections in people who have recovered from BA.5 infection. Our results suggest that BA.5 sub-variant is capable not only of bypassing humoral immunity boosted by SARS-CoV-2 infection, but also leads to a weak enhancement of humoral immunity itself. In contrast to our data, recent data indicated an enhanced neutralization against BA.5 following BA.5 infection in triple-vaccinated individuals (34). In the study by Wang et al. (34), serum samples were collected from already recovered patients at a mean of 32 days after infection, whereas in our study the sera were collected during the acute phase at hospitalization.

Interestingly, we found a weak IFN-γ response after stimulating PBMCs with selectively mutated regions of SARS-CoV-2 variants. One reason could be that the participants were still early in the infection and a measurable T-cell immunity against the mutated regions had not yet developed. Overall, all patient groups had a high positivity after cellular stimulation with S protein of Wuhan wildtype, regardless of vaccination status and variant responsible for breakthrough infection. PBMCs of patients with Omicron-BA.5 breakthrough infection showed the strongest IFN-γ response against Wuhan wildtype, followed by patients with Delta infection.

One limitation of this study are differences between cohorts regarding to demographic characteristics. For instance, among the BA.1-infected patients, 87.5% were under 70 years of age in the dually vaccinated group compared to 33.3% of boosted patients. That might be an explanation for the weaker humoral immune enhancement through BA-1 infection we observed in the group with booster vaccination. For instance, data has shown a reduced antibody neutralization response for elderly above 70 years after vaccination or infection (35, 36). Furthermore, the uninfected control group received a higher percentage of Spikevax (Moderna) vaccines than the patient groups, which could have influenced the results. However, studies found a similar high neutralization potential for individuals vaccinated with Spikevax (Moderna), Comirnaty (BioNTech/Pfizer) and a combination of vaccines (37).

In conclusion, we found strongly reduced neutralizing antibody titers against Omicron sub-variants BA.1 and BA.5. Furthermore, humoral immunity was boosted through Delta and Omicron-BA.1 infections in hospitalized double-vaccinated patients and patients with booster vaccination. This finding does not apply to BA.5 infections, in which we found no enhancing effect on humoral immunity. Despite BA.5 breakthrough infection, those patients may still be vulnerable for reinfections with BA.5 or other newly emerging SARS-CoV-2 variants. Further studies are needed to investigate the humoral and cellular immune response after breakthrough infection with BA.5 and its role in protecting from subsequent breakthrough infections.

## Data availability statement

The datasets presented in this study can be found in online repositories. The names of the repository/repositories and accession number(s) can be found below: PRJEB59607 (ENA; https://www.ebi.ac.uk/ena/browser/view/PRJEB59607).

## Ethics statement

The studies involving human participants were reviewed and approved by Ethik-Kommission der Medizinischen Fakultät der Universität Duisburg-Essen. The patients/participants provided their written informed consent to participate in this study.

## Author contributions

MB, LB, MA, MO, LT, LS, IK, AT, JG, PB, SC and MW performed the experiments. MB, LB, MA and MO were involved in sample collection. MB, AT, SD, UD, OW, FM and ML analyzed the data. AK, AS, HR and MB planned the study. AK and MB wrote the manuscript. All authors contributed to the article and approved the submitted version.
